# Morphological and molecular identification of *Eimeria rajasthani* (coccidia: Eimeriidae) in the dromedary camel (*Camelus dromedarius*) in Riyadh, Saudi Arabia

**DOI:** 10.3389/fvets.2024.1464138

**Published:** 2024-10-29

**Authors:** Esam M. Al-Shaebi, Saleh Al Quraishy, Sawsan A. Omer, Rewaida Abdel-Gaber, Osama B. Mohammed

**Affiliations:** Department of Zoology, College of Science, King Saud University, Riyadh, Saudi Arabia

**Keywords:** dromedary camels, coccidiosis, prevalence, taxonomy, morphology, genetic analysis

## Abstract

**Introduction:**

Coccidiosis is a serious parasitic disease in camels caused by an intestinal protozoan parasite of the genus *Eimeria*, which is linked to significant causes of reduced milk and meat production. In Saudi Arabia, scare literature focused on the coprological investigation of dromedary camels (*Camelus dromedarius*). To determine the taxonomic status of camel parasite species, we performed morphological characterization of oocysts and genetic analysis (*18S rRNA* and *ITS-1* gene regions) of *Eimeria* species collected from camels in Riyadh, Saudi Arabia.

**Methods:**

A total of 150 faecal samples were obtained from camels at the old camel market. These samples were tested for the presence of *Eimeria* oocysts using the conventional floatation technique before being sporulated in a 2.5% potassium dichromate solution. *Eimeria* oocysts were morphologically and molecularly examined and identified, and the infection rate of parasitic infections was determined.

**Results:**

Our findings revealed that the overall frequency of oocysts was 30%. The identified species was *Eimeria rajasthani*, which had a typical ellipsoidal oocyst shape. Oocystic polar granule, micropyle, micropylar cap, and oocyst residuum are not visible. Sporocysts are oval with stieda body. Sporocyst residuum contains many granules and sporozoites with refractile bodies and nuclei. Genetic analyses of the sequence data from the partial *18S rRNA* and *ITS-1* gene regions revealed that the sequences obtained from *E*. *rajasthani* oocysts are related to DNA sequences reported from *E. lamae* from the Alpaca from China, particularly the *18S rRNA* sequences.

**Conclusion:**

This study emphasized the need to use molecular phylogenetic tools to describe camel intestinal coccidian parasites with traditional morphology-based approaches to better understand their biology. For camel husbandry and disease control, more studies should be conducted to better understand the epidemiology of these protozoan parasites.

## Introduction

The dromedary camel, *Camelus dromedarius* (Order: Artiodactyla), is the most prevalent Camelidae species. Camels have been an essential animal in desert locations for ages due to their ability to tolerate severe conditions (high temperatures and drought), supply milk and meat, and serve as a means of transportation (1, 2). Camels are found in 35 countries around the world, 18 of which are African. According to recent official statistics, Saudi Arabia is home to approximately 1.8 million camels. Camels are prone to a variety of diseases, especially due to the lack of sufficient veterinary services (3). Gastrointestinal parasites are one of the most common challenges facing the global camel population (4), causing not only nutritional and immune deficiencies but also stunted growth and delayed development (5, 6). These parasitic infections affect camel production and the quality of their meat and milk (7–9).

*Eimeria* species are gut-dwelling intracellular coccidian parasites that spread by the fecal–oral pathway; non-sporulated oocysts are discharged in feces of infected animals (10). Sporulation of oocysts occurs over 2–7 days, depending on coccidian species and environmental factors (e.g., oxygen, temperature, and moisture) (11). Five *Eimeria* species are thought to have the capability of infecting the camel’s intestine (5). *Eimeria cameli* (12) and *Eimeria dromedarii* (13) are the most widely distributed species of camelid *Eimeria*, while others [*Eimeria bactriani* (14); *Eimeria rajasthani* (15); and *Eimeria pellerdyi* (16)] are found in specific geographical zones. Coccidiosis is most commonly reported in young animals, but adults are resistant due to an immunological response to previous *Eimeria* exposure (17–19). Camels with severe *Eimeria* infections exhibit symptoms such as hemorrhagic enteritis and diarrhea, loss of appetite, dehydration, and increasing weight loss (20). Furthermore, the free movement of camels across borders could lead to the spread of parasitic diseases (21–23).

*Eimeria* species have been identified using the shape of the sporulated oocysts and sporocysts (24). *Eimeria* species were identified using morphological features such as size, shape, color, sporulation time, texture of oocyst wall, presence or absence of micropyle, and micropylar cap, as well as (25) taxonomy keys. However, only a few *Eimeria* species have morphological resemblance with one another. Molecular analysis is required to reliably define *Eimeria* species and establish evolutionary relationships between them (26). Few studies have focused on the ability to use the internal transcribed spacer (ITS) region to identify camelid’s *Eimeria* species (27, 28). The previous studies in Saudi Arabia had addressed the phylogenetic relationships of coccidian species based on the ability of the use target genetic regions, including the small subunit ribosomal RNA (*18S rRNA*), internal transcribed spacer (*ITS*)-1, and mitochondrial cytochrome c oxidase I (*COI*) genes in identification and taxonomy of *Eimeria* species, which parasitize rodents (29), rabbits (30), sheep (31), broiler chicken (32), and domestic pigeons (33).

Several investigations on camelid coccidian infection have been conducted in Saudi Arabia (17–19, 34–36). Three protozoan parasites, namely, *Eimeria dromedarii, E. rajasthani*, and *E. cameli*, were detected in the dromedary camel in Saudi Arabia. The pathology of the three species has been evaluated, and they are pathogenic in young camels causing enteritis as a result of the intestinal mucosa destruction whereas older camels did not show clinical signs (17).

Similarly, to control coccidiosis in camels successfully and economically, an extensive understanding of the *Eimeria* species implicated is required. Therefore, the purpose of this study was to morphologically identify camelid *Eimeria* species and molecularly corroborate their classification.

## Materials and methods

### Fecal sample collection

A total of 150 fecal samples (10 g/animal) were collected, between January and April 2024, from dromedary camels in the old camel market in Riyadh (Saudi Arabia). These samples were obtained directly from the rectum using disposable gloves, placed into screw-capped plastic containers, and labeled with epidemiological data. The samples were then transported in an icebox to the Laboratory of Parasitology Research (Department of Zoology, College of Science, King Saud University) for further analysis.

### Coprological examination

All fecal samples were subjected to a floatation technique using a saturated saline solution (Sheather’s solution, specific gravity = 1.28) as reported by Soulsby (37). In brief, 3 g of fecal material from each sample was weighed, mixed with 15 mL of saturated sucrose solution, and homogenized. The fecal suspension was then centrifuged at 1,500 rpm for 3 min at room temperature (RT). The samples were examined using a light microscope (Olympus Corporation, Tokyo, Japan). To identify the species, positive samples with *Eimeria* oocysts were cultivated in Petri dishes containing 2.5% (w/v) potassium dichromate (Sigma-Aldrich) and incubated at 26 ± 2°C for 2–7 days until sporulation was achieved (38). After sporulation, the oocysts were washed three times in 1× phosphate-buffered saline (PBS) and kept at 4°C for further investigation. Photographs of oocysts (non-sporulated and sporulated) were acquired with a Leica DM 2500 microscope (NIS ELEMENTS software, version 3.8). The size and shape index of oocysts and sporocysts were calculated using ImageJ 1.53e software (Wayne Rasband and contributors, National Institute of Health, United States). The length, width, and shape index of the oocysts and sporocysts were measured for parasite species. Data were presented in micrometers (μm) as the mean, with the range in parentheses.

### Molecular analysis

DNA was isolated from *Eimeria* oocysts via a commercial QIAamp DNA Stool Mini Kit (Qiagen, Hilden, Germany) according to the manufacturer’s protocol. The concentration and purity of the genetic sample were evaluated using a NanoDrop ND-1000 spectrophotometer (Thermo Fisher Scientific, Inc., Wilmington, DE, United States). PCR was performed under conditions that targeted the partial *18S rRNA* and *ITS-1* gene regions. Amplification was carried out utilizing the genus-specific primers as follows: for the *18S rRNA* gene region was 5′-TAC CCA ATG AAA ACA GTT T-3′ and 5′-CAG GAG AAG CCA AGG TAG G-3′ (39), and the *ITS-1* gene region was 5′-GCA AAA GTC GTA ACA CGG TTT CCG-3′ and 5′-CTG CAA TTC ACA ATG CGT ATC GC-3′ (40). The reaction conditions were as follows: initial denaturation at 94°C for 2 min, then denaturation at 94°C for 50 s, annealing at 50°C (*18S rRNA*), and 52°C (*ITS-1*) for 30 s, and extension at 72°C for 30s in 35 cycles. PCRs were carried out using a Multigene™ thermocycler (Labnet International, Inc., NJ, United States). Amplified products were electrophoretically analyzed using a 1.5% (w/v) agarose gel (Sigma-Aldrich, United States) in 1 × Tris–boric acid–EDTA (TBE) and stained with SYBR Safe DNA gel dye (Thermo Fischer Scientific, Canada) and using Easy Ladder 1 (100 bp to 2000 bp) from Bioline, United Kingdom, as a molecular weight marker, indicating the size of the PCR products resulted from using these primers. Products were visualized using a gel documentation system (Image Analyzer, United Kingdom). The PCR products were sequenced using the Sanger dideoxy method available from Macrogen^®^ (Seoul, South Korea). Both *18S rRNA* and *ITS-1* regions were selected for easy comparison with related sequences in GenBank. Sequences were deposited at a public sequence database, GenBank of NCBI.^1^ The sequence homology was analyzed in GenBank using the BLASTn search.^2^ Data were aligned using CLUSTAL-X software (41). MEGA X software (42) was used to conduct maximum likelihood (ML) and neighbor-joining (NJ) analyses with the best-fit substitution models. Statistical support for each node was evaluated using a non-parametric bootstrap test with 1,000 replicates. Trees were drawn to scale, with branch lengths in the same units as those of the evolutionary distances used to infer the phylogenetic tree.

## Results

**Figure 1:**
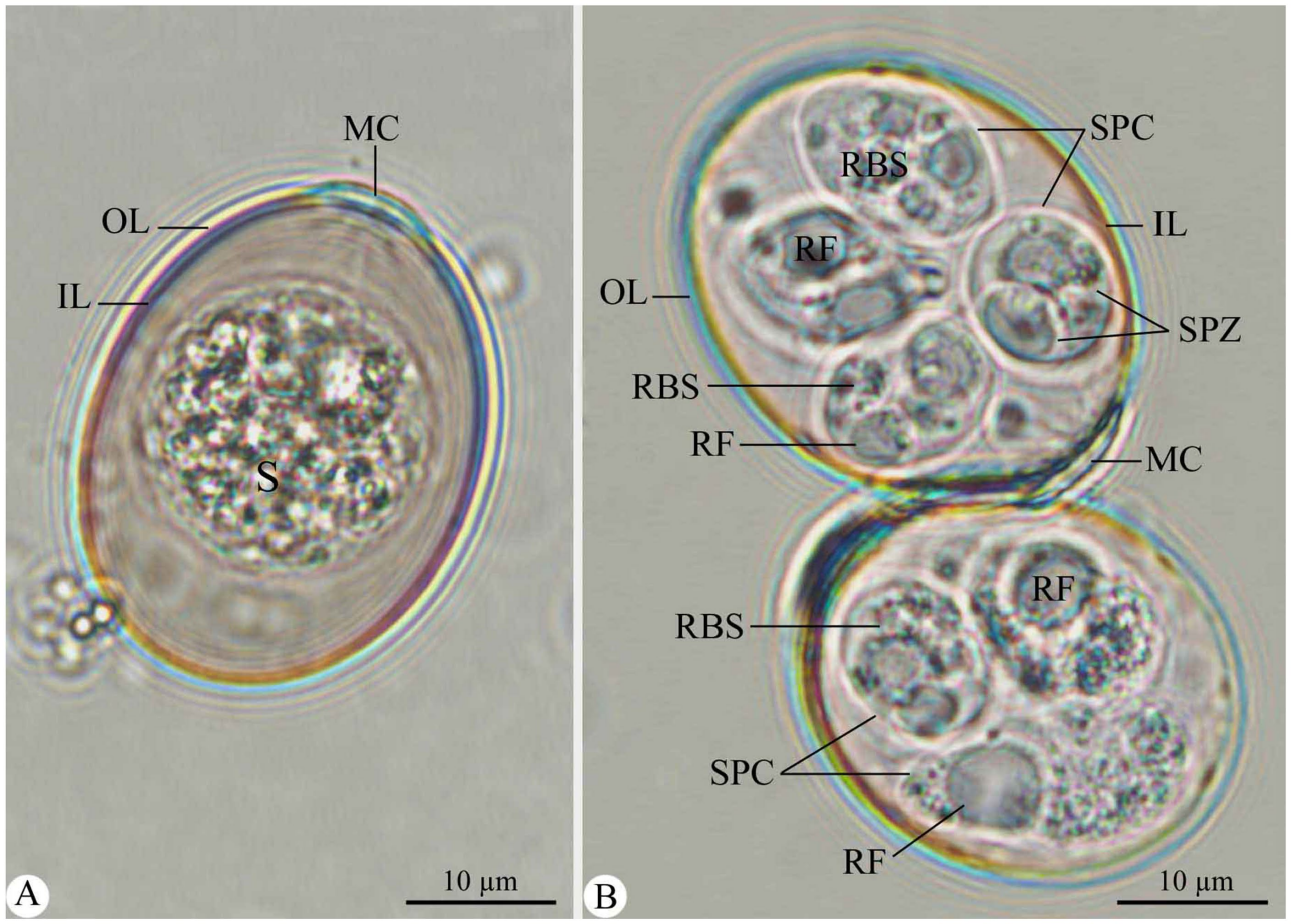
Morphological features of *E. rajasthani* oocysts infecting camels. (A) Non-sporulated *Eimeria* oocyst. (B) Sporulated *Eimeria* oocysts. OL, outer layer; IL, inner layer; S, sporont; MC, micropylar cap; SPC, sporocyst; SPZ, sporozoite; RF, refractile body; RBS, sporocyst residuum. Scale bar = 10 μm.

Out of the 150 examined fecal samples, 45 (30%) were infected with eimerian parasites. The recovered parasite possesses a unique taxonomic affinity for the genus *Eimeria*, particularly for *E. rajasthani*, as detailed below. Figure 1 depicts the oocysts of *Eimeria* species recovered from camels during the current study. Table 1 summarizes the morphometric parameters of the recovered *Eimeria* species.

**Table 1:** Morphological characteristics of sporulated oocysts for *E. rajasthani* from *Camelus dromedarius*.

References of *E. rajasthani*	Oocyst size				Oocyst shape	Sporocyst size			Locality
	Size	Micropyle	Polar granule	Oocyst residuum		Size	Sporocyst residuum	Stieda body	
Dubey and Pande (15)	34–39 (36) × 25–27 (25)	+ capped	−	−	Ellipsoidal	14–15 (15) × 8–11 (11)	+	+	India
Yagoub (46)	34–39.5 (35.6) × 26–29 (26.5)	+ capped	−	−	Ellipsoidal	12–16 (13.5) × 9–11 (9.9)	+	+	Eastern Region of Sudan
Mahran (47)	39.5 × 26	+ capped	−	−	?	?	?	?	Red Sea Governorate, Egypt
Metwally et al. (36)	25–30 × 21–24	+ capped	−	−	Oval	12–14 (13) × 9–10 (9.5)	−	?	Riyadh and Al-Qassim, Saudi Arabia
**Present study**	**27.86–37.42 (33.71) × 21.19–27.86 (25.61)**	**+ capped**	**−**	**−**	**Ellipsoidal**	**12.13–15.46 (13.97) × 8.64–11.58 (10.12)**	**+**	**+**	**Riyadh, Saudi Arabia**

+ = present; − = absent;? = not specified.

### Description

Non-sporulated oocysts are ellipsoidal, measuring 25.64–35.39 (32.15) in length and 20.20–25.65 (23.66) in width (Figure 1A). The oocyst wall is double-layered, with the outer one being thicker and inner one being membranous (Figure 1A). The micropyle is visible; the micropylar end has a dome-shaped micropylar cap, measuring 1.78–2.82 (1.99) in height and 7.63–10.53 (8.41) in width (Figure 1A). The sporont (zygote) is cylindrical, measuring 17.41–20.54 (20.31) μm × 17.44–20.84 (19.65) (Figure 1A). Sporulation took approximately 7 days at 27°C.

Sporulated oocysts are ellipsoidal, measuring 27.86–37.42 (33.71) in length and 21.19–27.86 (25.61) in width. Micropylar cap measures 7.63–10.53 (8.41) in width, whereas oocystic polar granule and oocyst residuum are absent (Figure 1B). Each oocyst was tetrasporozoic (Figure 1B). Sporocysts are oval, measuring 12.13–15.46 (13.97) in length and 8.64–11.58 (10.12) in width. They have a single-layered wall and Stieda body at the narrower end (Figure 1B). Sporocyst residuum exists between the two sporozoites (Figure 1B). Each sporocyst is dizoic. Sporozoites are elongated, lying longitudinally head to tail in the sporocysts, 10.84–12.83 (11.96) μm × 3.12–4.85 (4.10) μm, with one end broad and the other narrower but pointed and having two or more conspicuous globules (Figure 1B). Each sporozoite has one refractile body at the wider end (Figure 1B).

### Molecular analysis

The amplification of both *18S rRNA* and *ITS-1* gene regions for *E. rajasthani* was successful using primers that were used in the present study. The expected PCR products of ~613 bp and ~ 380 bp were obtained and sequenced for the *18S rRNA* and *ITS-1* gene regions, respectively. Four sequences were obtained from the *18S rRNA* region and deposited in GenBank and were given the accession numbers PP965651 to PP965654. Two sequences were obtained from the *ITS-1* region and were also deposited in GenBank and were given the accession numbers PP965655 and PP965656.

**Figure 2:**
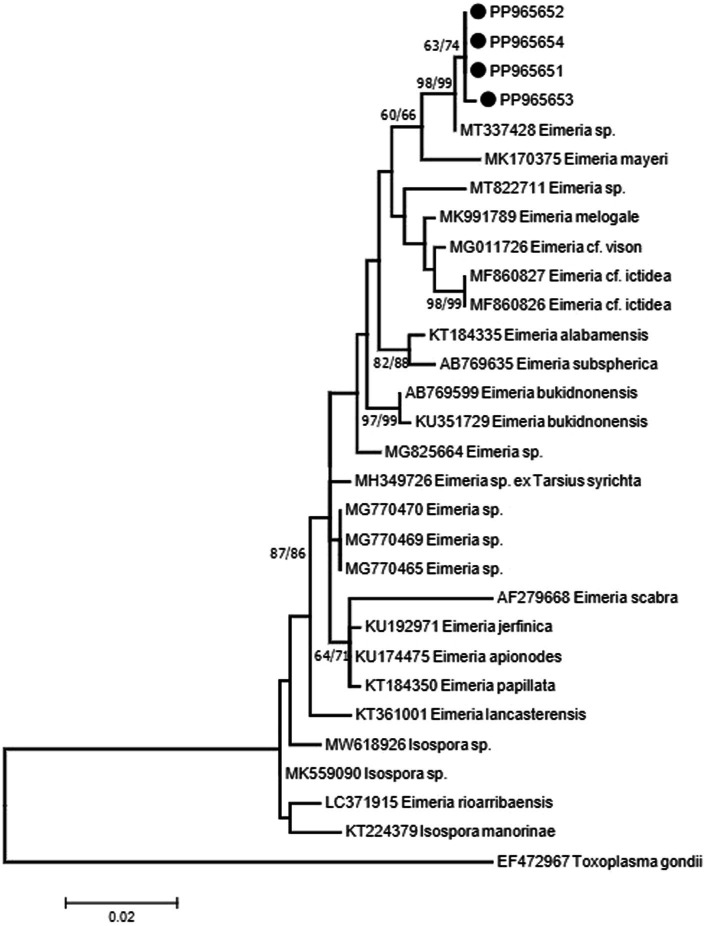
Consensus phylogenetic tree constructed with maximum-likelihood (ML) and neighbor-joining (NJ) methods, showing phylogenetic relationships between *E. rajasthani* (PP965651 to PP965654; shown on solid circles) and 25 related taxa obtained from NCBI GenBank with *Toxoplasma gondii* as an out-group. Numbers indicated at branch nodes are bootstrap values (ML/NJ). Only bootstraps >60% are shown.

The *18S rRNA* sequences showed two haplotypes with one sequence (PP965653) with a mutation (C/T) at position 238 on the alignment. Sequences showed 99% identity to sequence MT337428 isolated from the feces of Alpaca (*Vicugna pacos*) from China. The sequence from Alpaca showed one to two mutations when compared with sequences from *E. rajasthani* obtained in the present study. There is another sequence from the Alpaca MT337427 which was shorter than MT337428, which also showed identity to sequences obtained in the present study. Although the sequence did not cover the whole region studied, it has shown differences in three bases at positions 243, 245, and 280 of the alignment. The closest match for the sequences obtained in the present study other than MT337427 and MT337428 from the Alpaca was a sequence (MK170375) from *Eimeria mayeri* from Reindeer (*Rangifer tarandus tarandus*) from Norway with 97.8% identity. Phylogenetic analysis of the *18S rRNA* sequence data, resulting from neighbor-joining (NJ) and maximum-likelihood (ML) analyses, revealed that sequences from *E. rajasthani* and *Eimeria* sp. from the Alpaca shared a common ancestor and formed a monophyletic group (Figure 2). Sequences from the present study have shown 97% identity to several other eimerian sequences from carnivores and birds. Furthermore, it showed the same values from some isospora sequences from birds as it has been shown in Figure 2. Taxa used in the analysis are presented in Table 2.

**Table 2:** Taxa and their *18S rRNA* sequences GenBank accession numbers, their hosts, and their origin were used in the present analyses.

Accession number	Host	Country
**PP965651 *Eimeria rajasthani***	**Camel (*Camelus dromedarius*)**	**Saudi Arabia**
**PP965652 *Eimeria rajasthani***	**Camel (*Camelus dromedarius*)**	**Saudi Arabia**
**PP965653 *Eimeria rajasthani***	**Camel (*Camelus dromedarius*)**	**Saudi Arabia**
**PP965654 *Eimeria rajasthani***	**Camel (*Camelus dromedarius*)**	**Saudi Arabia**
MT337428_*Eimeria*_sp.	Alpaca (*Vicugna pacos*)	China
MK170375_*Eimeria_mayeri*	Reindeer (*Rangifer tarandus tarandus*)	Norway
MF860827_*Eimeria_cf._ictidea*	Black-footed Ferrets (*Mustela nigripes*)	Canada
MF860826_*Eimeria_cf._ictidea*	Black-footed Ferrets (*Mustela nigripes*)	Canada
MK991789_*Eimeria_melogale*	Javan ferret-badger (*Melogale orientalis*)	Czech Republic
MG011726_*Eimeria_cf._vison*	American Mink (*Neovison vison*)	Australia
KT184335_*Eimeria_alabamensis*	Cow (*Bos taurus*)	Canada
MG825664_*Eimeria*_sp.	Capercaillie grouse (*Tetrao urogallus*)	Poland
MT822711_*Eimeria*_sp.	Père David’s deer (*Elaphurus davidianus*)	China
MG770470_*Eimeria*_sp.	Shrew (*Crocidura* sp.)	Romania
MG770469_*Eimeria*_sp.	Shrew (*Crocidura* sp.)	Bulgaria
MG770465_*Eimeria*_sp.	Shrew (*Crocidura suaveolens*)	Czech Republic
KU192971_*Eimeria_jerfinica*	Striped Field Mouse (*Apodemus agrarius*)	Czech Republic
MH349726_*Eimeria_sp._ex_Tarsius_syrichta*	Philippine tarsier (*Carlito syrichta*)	Czech Republic
MW618926_*Isospora*_sp.	Northern Flicker (*Colaptes auratus luteus*)	Canada
MK559090_*Isospora*_sp.	Eurasian Wren (*Troglodytes troglodytes*)	Czech Republic
LC371915_*Eimeria_rioarribaensis*	Northern Bat (*Eptesicus nilssonii*)	Japan
AF279668_*Eimeria_scabra*	Pig (*Sus domesticus*)	Germany
KT224379_*Isospora_manorinae*	Yellow-throated miner (*Manorina flavigula wayensis*)	Australia
KT361001_*Eimeria_lancasterensis*	Eastern Gray Squirrel (*Sciurus carolinensis*)	Czech Republic
AB769635_*Eimeria_subspherica*	Cow (*Bos taurus*)	Japan
KU174475_*Eimeria_apionodes*	Yellow-necked mouse (*Apodemus flavicollis*)	Czech Republic
KT184350_*Eimeria_papillata*	House Mouse (*Mus musculus*)	Canada
AB769599_*Eimeria_bukidnonensis*	Cow (*Bos taurus*)	Japan
KU351729_*Eimeria_bukidnonensis*	Cow (*Bos taurus*)	Turkey
EF472967_*Toxoplasma_gondii*	RH Strain	China

Sequences from *E. rajasthani* reported in the present study are shown in bold.

The *ITS-1* sequences (PP965655 and PP965656) from *E. rajasthani* have shown 94–95% to *E. lamae* (GQ330537 {USA}, MW838990 {China}, MW838989 {China}), the only three sequences available in GenBank. The two sequences obtained in the present study showed two haplotypes with a mutation at position 136 (C/G) on the alignment.

## Discussion

Infection with coccidian intestinal parasites has a significant economic impact because of losses due to enteritis, diarrhea, and decreased body weight in camels, which also affects meat yield and quality (43). There is little information available on the epidemiology of coccidian intestinal parasites in dromedary camels in Saudi Arabia. As a result, the purpose of this study was to conduct coprological and molecular investigations of camelids *Eimeria* species to provide additional information about these parasites in the Riyadh region, Saudi Arabia. In the current study, the infection rate with *Eimeria* species in dromedary camels was 30%. Several studies have revealed infection rates in various camel-rearing regions across the world. In earlier studies, Kawasmah and El Bihari (35), Kasim et al. (18), and Hussein et al. (17) discovered one or more species (*E. rajasthani*, *E. cameli*, and *E. dromedarii*) with an overall prevalence of 14, 41.6, and 40% in Saudi Arabian camels, respectively. Mahmoud et al. (19) found a mean infection rate of 15.7% for adult camels and 10.2% for camel calves in Saudi Arabia’s central region. Metwally et al. (36) investigated coccidiosis in camels in Saudi Arabia and discovered that the prevalence of *Eimeria* oocysts in Riyadh was 33.89% and in Al-Qassim 38.46%. According to Sazmand et al. (44), changes in the prevalence of coccidian infections in camels are likely due to environmental and host-related factors.

Different diagnostic methods for *Eimeria* species are currently available, with varying degrees of specificity and sensitivity, including morphological examination and DNA molecular tools (45). There are five recognized old-world camelid eimerian species (including *E. cameli*, *E. dromedarii*, *E. bactriani*, *E. rajasthani*, and *E. pellerdyi*). Based on the morphological findings, the species detected in the camel in Riyadh (Saudi Arabia) is related to *E. rajasthani*. The main criteria for identifying recovered *E. rajasthani* were the ellipsoidal shape of oocysts and the presence of a dome-shaped micropylar cap. Our descriptions of the sporulated oocysts of *E. rajasthani* were similar to those of Dubey and Pande (15), Yagoub (46), Mahran (47), and Metwally et al. (36). Although Metwally et al. (36) described the oocysts of *E. rajasthani* as oval, they did not demonstrate the oocyst residuum and the oocyst Stieda body.

Five eimerian species have also been described from the New World camelids which are as follows: *E. macusaniensis, E. lamae, E. alpacae, E. punoensis, and E. ivitaensis* (48). *E. rajasthani* showed close similarity in measurements with *E. lamae* from the Alpaca (*V. pacos*). However, the shape of the micropylar cap is different between the two organisms. There was no association between Alpacas and the dromedary camel in the present study; therefore, it is unlikely that the species of *Eimeria* detected in the present study could be *E. lamae*. Furthermore, *E. lamae* has never been reported from Saudi Arabia.

According to Ipczynski (49), Hussein et al. (17), and Dia et al. (50), *E. dromedarii*, *E. rajasthani*, and *E. cameli* are more pathogenic species to young camel calves; thus, the presence of these three pathogenic *Eimeria* species indicated that coccidiosis could be contributing to enteric syndromes in camels. Yagoub (46) described a clear identification of *E. dromedarii* and *E. cameli*, which may be utilized to distinguish the recovered *E. rajasthani* from them. In this study, the oocysts of *E. rajasthani* are distinct from *E. cameli* on account of the shape of the oocyst (vs. truncate ovoid in *E. cameli*), sporocyst (vs. elongated in *E. cameli*), and sporozoites (vs. comma-shaped in *E. cameli*), the smaller size of both oocyst (vs. 86.6 × 66.2 μm in *E. cameli*) and sporocyst (vs. 37.4 × 18.61 μm in *E. cameli*), bilayered oocyst wall (vs. three-layered in *E. cameli*), the presence of micropyle with 17.3–26.0 μm in width as well as polar granule in *E. cameli*, and 7 days for sporulation (vs. 12–15 days in *E. cameli*). The eimerian oocysts from the present study differ from those of *E. dromedarii* due to the larger size of both oocysts (vs. 28.1 × 23.4 μm in *E. dromedarii*) and sporocysts (vs. 9.0 × 7.3 μm in *E. dromedarii*), their different oocyst shape (subspherical to ovoid shape in *E. dromedarii*) and sporozoites (vs. ovoid in *E. dromedarii*), the presence of micropyle as well as Stieda body in *E. dromedarii*, and the absence of sporocyst residual in *E. dromedarii*.

Furthermore, Prasad (16) provided a detailed description of *E. pellerdyi*, which was utilized to compare with the recovered *E. rajasthani*. The recovered *E. rajasthani* oocysts differ from *E. pellerdyi* in terms of oocyst shape (vs. ovoidal in *E. pellerdyi*) and sporozoites (vs. club-shaped in *E. pellerdyi*), the smaller size of its oocyst (vs. 22.5–24 × 12–13.5 μm in *E. pellerdyi*) and sporocyst (vs. 4.5–6 × 9–10.5 μm in *E. pellerdyi*), the absence of a micropylar cap, and 7 days for sporulation (vs. 5 days in *E. pellerdyi*). Furthermore, Utebaeva et al. (10) described *E. bactriani* in detail, and their data were used to compare it to the recovered *E. rajasthani*. *Eimeria* oocysts differ from *E. bactriani* in the shape of oocyst (vs. spherical in *E. bactriani*), sporocyst (vs. lemon-shaped in *E. bactriani*), and sporozoites (vs. pear-shaped in *E. bactriani*), larger oocyst size (vs. 29.1 × 26.6 μm in *E. bactriani*), and indistinct micropyle (vs. observed in *E. bactriani* with 5–7 μm width).

Our findings are regarded as a re-description of the discovered camelid’s *E. rajasthani* parasite in Saudi Arabia, with adequate morphological and morphometric data. Molecular characterization has recently gained popularity for assuring accurate *Eimeria* species identification, especially when morphological differentiation is problematic due to shape and size similarities (32).

The *18S rRNA* sequences obtained from oocysts of *E. rajasthani* showed 99% sequence similarity to those from *Eimeria* sp. from the Alpaca (MT337428) from China, which was later described as *E. lamae* by Gao et al. (51). The phylogenetic tree generated from the *18S rRNA* sequence data indicated that both *E. lamae* and *E. rajasthani* shared a common ancestor. Another sequence from *E. lamae* (MT337427) reported by Gao et al. (51) was shorter; however, it showed identity to sequences from *E. rajasthani* and MT337428. The similarity of *E. rajasthani* and those from reindeer and carnivores raises a question about the origin and evolution of *E. rajasthani*.

*ITS-1* sequences reported from *E. rajasthani* have shown 94–95% identity to sequences from *E. lamae* (51). There were no available sequences for the same region at GenBank; therefore, it was not possible to generate a phylogenetic tree from the available data.

It was proposed by Hnida and Duszynski (52) that eimerian parasites from rodents with a sequence of ≤5% at the *ITS-1* region could support conspecific types which are morphologically similar, whereas differences of >5% in the same region may be used to resolve separate species of *Eimeria*. This suggestion was further supported by Motriuk-Smith et al. (53) who studied genetic variation in squirrels (*Sciurus niger*). It has also been added that the *ITS-1* marker must be used cautiously, and it must be supplemented by other markers together with morphometric data (52, 54).

Morphological and morphometric data of *E. rajasthani* detected from the dromedary camel indicated a close resemblance to *E. lamae* from the New World camelid, the Alpaca from China. In addition, molecular data from the 18S rRNA sequences from *E. rajasthani* showed the identity of 99% to those of *E. lamae* as well; however, there was 95% identity to sequences from the *ITS-1* region of both sequences. The identity of the organism we are dealing with from the dromedary camel is certainly *E. rajasthani*, as there is no possibility that it has been acquired from another species other than the dromedary camel and there is no mixture between the dromedary camel and Alpacas in Saudi Arabia. From the present results, in particular the *ITS-1* data results, it is tempting to suggest that *E. rajasthani* and *E. lame* are conspecific. However, further study is required on different genes, particularly a mitochondrial gene such as cytochrome oxidase I (*COI*), to support this assumption and resolve the taxonomic status of each of *E. rajasthani* and *E. lamae*.

## Conclusion

This study provides further understanding regarding *E. rajasthani* oocysts infecting its type host (*C. dromedarius*) from Riyadh (Saudi Arabia) by combining a morphological description of oocysts and a genetic analysis. Furthermore, the GenBank database currently includes unique genetic sequences for the target gene regions retrieved from this coccidian species. Further studies are recommended to incorporate preventative and control approaches to combat *E. rajasthani* infection in the dromedary camel in Saudi Arabia.

## Data Availability

The datasets presented in this study can be found in online repositories. The names of the repository/repositories and accession number(s) can be found at: https://www.ncbi.nlm.nih.gov/genbank/, PP965651, PP965652, PP96563, PP965654, PP965655, PP965656.

## Footnotes

^1^ https://www.ncbi.nlm.nih.gov/

^2^ https://blast.ncbi.nlm.nih.gov/Blast.cgi
